# Potential Advances of Adjunctive Hyperbaric Oxygen Therapy in Infective Endocarditis

**DOI:** 10.3389/fcimb.2022.805964

**Published:** 2022-02-03

**Authors:** Christian Johann Lerche, Franziska Schwartz, Mia Marie Pries-Heje, Emil Loldrup Fosbøl, Kasper Iversen, Peter Østrup Jensen, Niels Høiby, Ole Hyldegaard, Henning Bundgaard, Claus Moser

**Affiliations:** ^1^ Department of Clinical Microbiology, Copenhagen University Hospital, Rigshospitalet, Copenhagen, Denmark; ^2^ Department of Virus and Microbiology Special Diagnostics, Statens Serum Institut, Copenhagen, Denmark; ^3^ Department of Cardiology, Rigshospitalet, Copenhagen University Hospital, Copenhagen, Denmark; ^4^ Department of Cardiology, Herlev and Gentofte Hospital, University of Copenhagen, Herlev, Denmark; ^5^ Department of Emergency Medicine, Herlev and Gentofte Hospital, University of Copenhagen, Herlev, Denmark; ^6^ Department of Immunology and Microbiology, Costerton Biofilm Center, Faculty of Health and Medical Sciences, University of Copenhagen, Copenhagen, Denmark; ^7^ Department of Anaesthesia, Rigshospitalet, University of Copenhagen, Copenhagen, Denmark

**Keywords:** hypoxia, biofilm, neutrophils, host response, inflammation, cytokines, reactive oxygen species

## Abstract

Patients with infective endocarditis (IE) form a heterogeneous group by age, co-morbidities and severity ranging from stable patients to patients with life-threatening complications with need for intensive care. A large proportion need surgical intervention. In-hospital mortality is 15-20%. The concept of using hyperbaric oxygen therapy (HBOT) in other severe bacterial infections has been used for many decades supported by various preclinical and clinical studies. However, the availability and capacity of HBOT may be limited for clinical practice and we still lack well-designed studies documenting clinical efficacy. In the present review we highlight the potential beneficial aspects of adjunctive HBOT in patients with IE. Based on the pathogenesis and pathophysiological conditions of IE, we here summarize some of the important mechanisms and effects by HBOT in relation to infection and inflammation in general. In details, we elaborate on the aspects and impact of HBOT in relation to the host response, tissue hypoxia, biofilm, antibiotics and pathogens. Two preclinical (animal) studies have shown beneficial effect of HBOT in IE, but so far, no clinical study has evaluated the feasibility of HBOT in IE. New therapeutic options in IE are much needed and adjunctive HBOT might be a therapeutic option in certain IE patients to decrease morbidity and mortality and improve the long-term outcome of this severe disease.

## Highlights

- Infective endocarditis (IE) is considered a biofilm infection of the endothelium of the heart, mainly localized to the heart valves. IE also include infection related to devices implanted in the heart.
- Potential beneficial effects of Hyperbaric Oxygen Treatment (HBOT) in bacterial infections:
  - Decreased tissue hypoxia
  - Reduced biofilm formation
  - Enhanced beneficial host responses
  - Reduced pro-inflammatory cytokines and adhesins
  - Enhanced growth factors and anti-inflammatory cytokines
  - Enhanced microbial killing of oxygen-dependent antibiotics
  - Reduced microbial growth and virulence (i.e. toxin production)
- An improved understanding of the mechanisms of host-pathogen interactions will help optimize personalized treatment and explore HBOT as a new adjunctive treatment strategy in IE

## Introduction

Infective endocarditis of native as prosthetic valves can be characterized as a biofilm infection. The diagnosis and treatment can be complicated and prolonged antibiotic treatment is needed to eradicate the infection. The antibiotic bioavailability, penetration and efficacy in a biofilm infection as IE are varying and often combination treatment is needed for a successful outcome. Especially, in complicated IE with treatment failure, prosthetic valves, aortic rod abscess and transcatheter aortic valve implantation (TAVI) devices where reoperation are difficult new treatment options are much needed. Adjunctive hyperbaric oxygen therapy (HBOT) could be a potential supplement to antibiotic treatment to enhance the antibiotic efficacy and host response against infection.

HBOT is administered as primary or adjunctive treatment in various non-infectious and infectious diseases (Mathieu et al., 2017; Moon, 2019). In this review, we highlight the potential beneficial aspects of adjuvant HBOT based on the pathogenesis in infective endocarditis (IE) and the known mechanisms of HBOT in various inflammatory diseases. Currently, necrotizing soft tissue infections (NSTI) including clostridal myositis (gas gangrene), chronic refractory osteomyelitis and brain abscess are conditions approved for adjunctive HBOT by the Undersea and Hyperbaric Medical Society (UHMS), federal drug administration (FDA) in the USA, and by the European Underwater and Baromedical Society (EUBS) (Mathieu et al., 2017; Moon, 2019). HBOT is also approved for crush injury, chronic diabetic and ischemic wounds, acute thermal burn injuries and compromised skin grafts and flaps (Moon, 2019), which often is complicated by bacterial biofilm (Malone et al., 2017). The generally accepted indications and level of evidence for the use of HBOT are systematic reviewed (Mathieu et al., 2017).

IE is defined as an infection of the endocardium, most prevalent on the heart valves (native or prosthetic) or on implanted cardiac devices (Moreillon and Que, 2004). The incidence of IE is increasing (Jensen et al., 2021; Talha et al., 2021) and without treatment the mortality rate is 100%. Even with current standard treatment the mean in-hospital mortality is as high as 15–20% (Iversen et al., 2019; Pries-Heje et al., 2021), 1-year mortality is up to 30-40% (Abegaz et al., 2017) and approximately 50% need valve surgery during the acute disease-course (Habib et al., 2019). IE is a serious acute infection characterized by bacterial vegetation formation composed of endothelial cells, platelets, monocytes and neutrophils, as well as extracellular components including fibrin, fibrinogen and collagen. Bacterial adherences to the damaged endothelial cells or sterile vegetation promote a cascade of interactions between host cells and colonizing pathogen (Werdan et al., 2014; Moser et al., 2017; Lerche et al., 2021).

A hallmark of IE is the pathogen-host interactions causing biofilm formation of the endocardium or prosthetic material. Besides the local bacterial colonization, the disease is often complicated by bacterial dissemination to various organs (brain, spleen, kidney and liver), which may cause peripheral abscess formation and inflammation – or embolization-induced tissue hypoxia (Moser et al., 2017). *Staphylococci*, *streptococci*, and *enterococci* are the causative pathogens in >80% of patients with IE (Moreillon and Que, 2004). The leading pathogen is *Staphylococcus aureus* (*S. aureus*) in high income countries followed by viridans and oral streptococci and *E. faecalis* (McDonald et al., 2005). Especially, in left-sided IE virulent pathogens such as *S. aureus and Streptococcus species* can subsequently progress in to a hypercoagulable state, septic shock, meningitis and multiple organ dysfunction (Lerche et al., 1995; Mourvillier et al., 2004; Fernández Guerrero et al., 2012). The colonization of the valves and septic state may trigger an exaggerated host response, by excessive chemotaxis of platelets, monocytes and neutrophils to the site of inflammation, which may lead to additional tissue and organ damage (Hamzeh-Cognasse et al., 2015; Hsu et al., 2019).

Neutrophils represent the primary defense against invading bacterial pathogens, however, the penetration and efficacy of neutrophils in valve vegetations are limited (Durack and Beeson, 1972b). The neutrophils may instead contribute to vegetation expansion (Hsu et al., 2019), and activate complex interactions with platelets and endothelial cells (Jung et al., 2015; Folco et al., 2018; Zucoloto and Jenne, 2019), host adhesins and pathogen adhesins and secreted extracellular proteins (Shun et al., 2005; Binsker et al., 2018). The availability of oxygen (O_2_) in the infected tissues is crucial, as many important cellular immune functions are dependent on oxygen availability for optimal cellular processes. To function optimally during infection of the host, the neutrophils needs an abundance of oxygen to drive the respiratory (oxidative) burst to ensure efficient cytotoxic killing of the pathogens, especially in the microenvironment of deep-sited infections and biofilms characterized by tissue hypoxia.

Moderate to severe anemia is prevalent in almost 50% of patients with IE, which might contribute to tissue hypoxia (Pries-Heje et al., 2021). HBOT has recently been shown to increase hemoglobin levels (Bosco et al., 2021) another immunomodulatory effect that could be beneficial for improving the long-term outcome of IE patients.

Two preclinical (animal) studies have shown beneficial effect of HBOT in IE (Özkan et al., 2016; Lerche et al., 2017), but so far, no clinical study has evaluated the feasibility of HBOT in IE and only a single case reporting a beneficial outcome (Chen et al., 2021).

## Administration of HBOT and Effect on Oxygen Delivery to Tissues

HBOT is a clinically used treatment modality in which a person breathes pure oxygen (100%) under an increased atmospheric pressure most commonly applied for sessions of 1-1.5 hours (typically once or twice a day). For clinical purposes, the common treatment protocol involves breathing ~100% oxygen at a pressure of 2.0–2.8 atmosphere absolute [ATA] (203–284 kPa) corresponding to diving at 10–18 m below sea level. HBOT can be carried out in either a mono- (single person) or multi-place (typically 2 to 14 patients) pressure chamber. In the blood, ~98% of the present oxygen is bound to the erythrocytes at atmospheric pressure and approximately 2% is dissolved in the plasma. Due to the high fraction of oxygen bound to erythrocytes a further increase in the oxygen tension may only be achieved through an increase in free dissolved oxygen (Henry´s law). Thus, HBOT increases the amount of dissolved oxygen in the plasma and increases the oxygen delivery in tissues, independently of hemoglobin (Gill and Bell, 2004). In a normal healthy person the oxygen tension in arterial blood is 80–100 mmHg, in venous blood 30–40 mmHg, in tissues about 60 mmHg (Kanick et al., 2019) and in uninfected bone 40-50 mmHg and in infected bone 10-20 mmHg (Mader et al., 1980). During HBOT treatment, the arterial oxygen tension exceeds 2,000 mmHg (at 3 ATA) (6.8 ml O_2_/100 ml of blood) and levels of 200–400 mmHg in tissues (Thom, 1989). This means that the availability of free oxygen increases ~20 fold in arterial blood, ~10 fold in venous blood and ~6-10 fold in the tissue during HBOT (2.8 ATA).

## Mechanisms of Action Related to Infection

**Figure 1:**
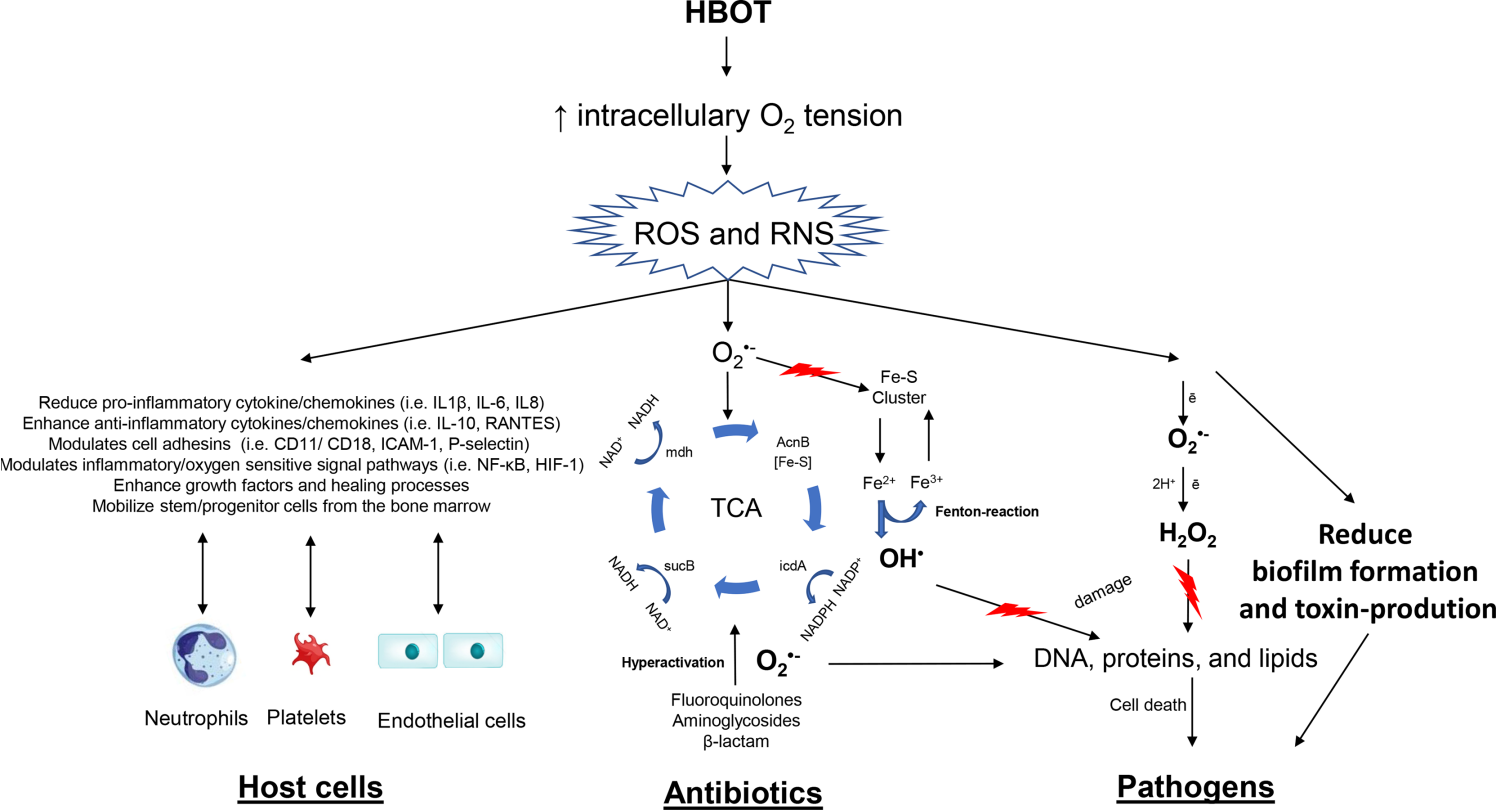
Overview on therapeutic mechanisms of hyperbaric oxygen (HBOT). The figure outlines effects that occur due to increased production of reactive oxygen species (ROS) and reactive nitrogen species (RNS) because of hyperoxia. Oxygen-dependent antibiotics stimulate oxidation of NADH *via* the electron transport chain that is dependent upon the tricarboxylic acid (TCA) cycle. Hyperactivation of the electron transport chain stimulates superoxide formation. Superoxide damages iron-sulfur clusters, making ferrous iron available for oxidation by the Fenton reaction. The Fenton reaction leads to hydroxyl radical formation, and the hydroxyl radicals damage DNA, proteins, and lipids, which results in lethal bacterial killing. This mechanism is further enhanced by HBOT potentiating antibiotics. HBOT has a direct effect on pathogens increasing reducing growth rates and toxin-production. The important immunomodulatory effects of HBOT on neutrophils, platelets and endothelial cells are illustrated.

Increased tissue oxygenation by HBOT affects three major components of importance in infectious diseases: (1) The host cell function, (2) The oxygen-dependent killing of specific antibiotics, and (3) The direct effects on the pathogen (**Figure 1**).

The main effect of HBOT relates to the increased tissue oxygenation (hyperoxia). It is well accepted, that HBOT will increase the intracellularly production of reactive oxygen species (ROS) and reactive nitrogen species (RNS) in various cells (Jamieson et al., 1986; Thom, 1989) and neutrophils (Almzaiel et al., 2013). ROS includes superoxide anion ( ${\text{O}}_{2}^{-}$ ), peroxide ( ${\text{O}}_{2}^{-2}$ ), hydrogen peroxide (H_2_O_2_), hydroxyl radicals (OH), and hydroxyl (OH^−^) induced by hyperoxia and the neutrophil-mediated killing of pathogens. ROS are toxic to the pathogen causing damage to DNA, proteins and lipids. ROS and RNS serve as effector and/or signaling molecules in a number of pathways, transduction cascades and inducer of cytokines/chemokines, hormones and growth factors (Valko et al., 2007; Circu and Aw, 2008; Kemp et al., 2008; Thom, 2011).

## Hyperbaric Oxygen and Host Response

### Neutrophils – Oxygen-Independent and Oxygen-Dependent Effects

**Figure 2:**
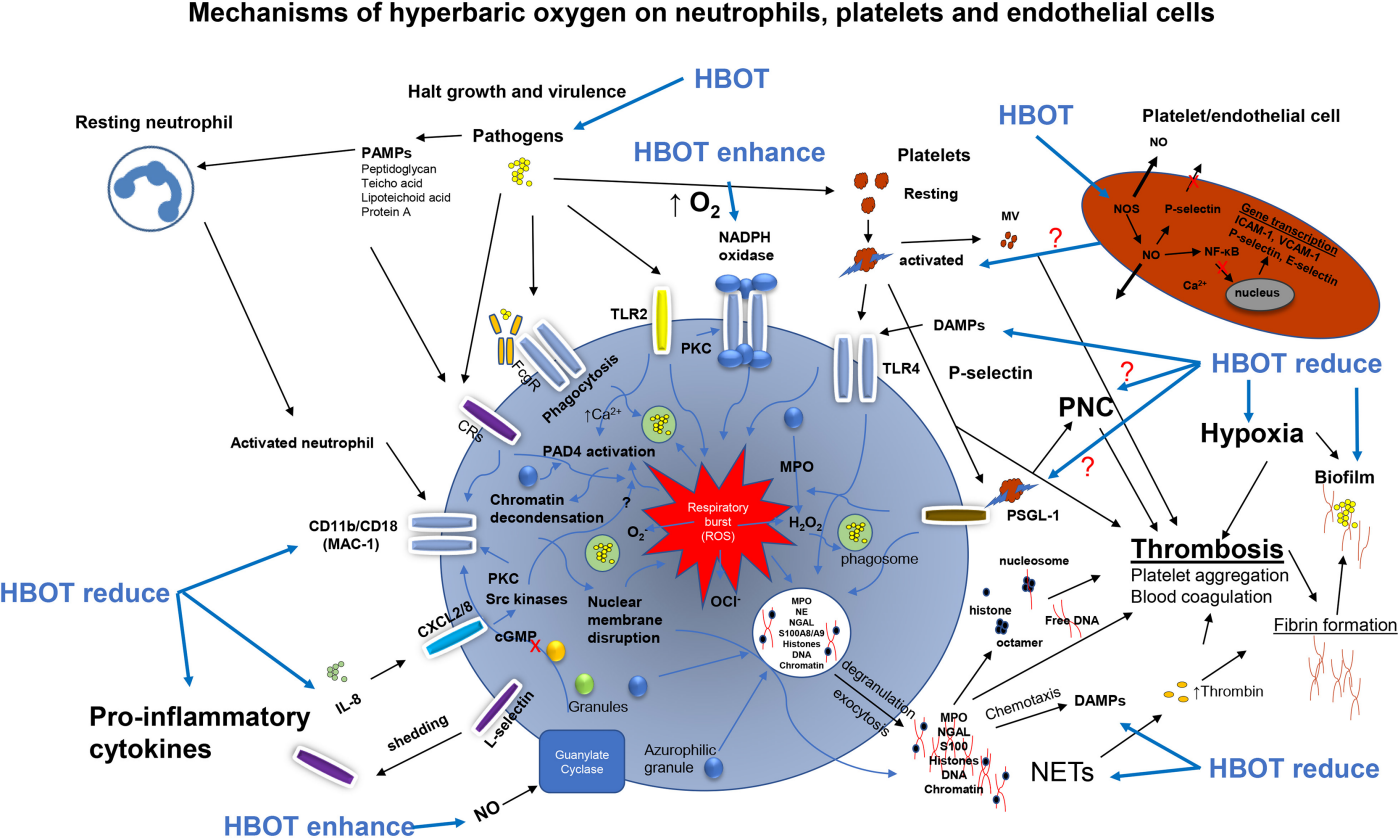
Overview of the activations pathways of neutrophils, platelets and endothelial cells promoted by pathogens and the possible effects of hyperbaric oxygen treatment (HBOT). Pathogens activates the NADPH oxidase driving the respiratory burst when phagocytized. Pathogens directly activates toll-like receptor 2 (TLR2 or TLR4) and complement receptors (CRs) enhancing the respiratory burst generating reactive oxygen species e.g. hydrogen peroxide (H_2_O_2_), superoxide (O_2^-^_) and hypochlorite (OCl^-^) for bacterial killing of phagocytosed bacteria. On the cell wall of bacteria are several pathogen-associated molecular patterns (PAMPs) which are pathogen derived alerting the host response. Host derived damage-associated molecular pattern (DAMPs) also alert the host response enhancing chemotactic signaling. The activated neutrophil upregulates the expression B_2_ integrin CD11b/CD18 (MAC-1) and integrin L-selectin released (shedding) by increased expression of CD11b/CD18. Platelets are directly activated by *S. aureus* and activated platelets bind toll-like receptor 4 (TLR4) stimulating ROS. Activated platelets expressing P-selectin bind to the ligand PSGL-1 forming neutrophil-platelets complexes (PNC) enhancing the respiratory burst system. Activated platelets and lysed platelets release platelet microvesicles (MV) in circulation activating the intrinsic pathway (contact pathway) of the coagulation cascade leading to increased thrombin. Excessive platelet binding of the PMN triggers neutrophil extracellular traps (NET) release and hyperactivation of the ROS system transporting granules to the membrane by exocytosis and NETosis (membrane disruption), exact mechanism unknown. Antimicrobial peptides are released (e.g. myeloperoxidase (MPO), neutrophil elastase (NE), neutrophil gelatinase-associated lipocalin (NGAL), S100A8/A9) to the extracellular space. Besides antimicrobial components NETs consist of chromatin, DNA and histone facilitating thrombosis and thrombin generation. Activation of NADPH oxidase results in the activation of protein-arginine deiminase 4 (PAD4) converting arginine to citrulline on histones and chromatin decondensation in the nucleus of the PMN. NE and MPO are release for granules (azurophilic) in the cytosol facilitating H_2_O_2_ and unfolding of chromatin. Interleukin 8 (IL-8) bind CXCL2/8 increasing CD11b/CD18 by activation of pathways (protein kinase C, PKC) and Src kinases) inducing NET release. Collectively, these activation pathways are believed to be halted by HBOT resulting in reduced collateral tissue damage in severe infections as infective endocarditis. NADPH, Nicotinamide Adenine Dinucleotide Phosphate; Protein Kinase A; ROS, Reactive Oxygen Species.

Neutrophils play a central role in the innate immune response and a critical role in bacterial killing (**Figure 2**). Neutrophils kill microorganisms by oxygen-independent and oxygen-dependent mechanisms (Elsbach et al., 1985). The ability of activated neutrophils to ingest and subsequently kill invading microbes is essential for the integrity of the host to maintain infection control. Neutrophils remove pathogens through phagocytosis. This engulfment of pathogens by neutrophils is oxygen-independent (Elsbach et al., 1985) and is possible under hypoxic conditions. However, the oxygen-independent system alone is inadequate to eradicate all pathogens and a decreased killing of pathogens is seen in a hypoxic environment (Mandell, 1974; Hunt et al., 1975; Hohn et al., 1976). Various studies have shown that the phagocytic activity improves during short-course of standard HBOT, for example in diabetic foot infections (Top et al., 2007) and in healthy volunteer divers (Labrouche et al., 1999) and *in vitro* (Almzaiel et al., 2013). Neutrophils may also contribute to depletion of oxygen (Kolpen et al., 2010; Jensen et al., 2019). Hypoxia selectively inhibits the respiratory burst activity and killing of *S. aureus* in human neutrophils (McGovern et al., 2011) and has also been shown in other chronic infections (Jensen et al., 2010).

The oxygen-dependent neutrophil respiratory burst, leads to the production of several different ROS (Babior et al., 2002; Quinn and Gauss, 2004; Van Der Veen et al., 2009). ROS have direct antimicrobial properties and also facilitate the degradation and destruction of pathogens by host cells (Johnson and Travis, 1979; Reeves et al., 2002). HBOT has been shown to enhance the respiratory burst killing mechanism of neutrophils. Mader et al. demonstrated the significance of increased oxygen tension to improve the neutrophils killing capacity against *S. aureus* in an *in vitro* assay of rabbit neutrophils and opsonin (Mader et al., 1980). Comparable findings have been reported by Almzaiel et al. in neutrophil-like cells (HL-60) compared to normoxia and hypoxia-treated cells (Almzaiel et al., 2013) *in vitro* and Labrouche et al. in circulating neutrophils isolated from divers (Labrouche et al., 1999), and Schwartz et al. in isolated human neutrophils (Schwartz et al., 2021). While these studies indicate that HBOT enhances the oxygen-driven respiratory burst of neutrophils leading to an increased bacterial killing, other studies have reported no effect of HBOT on the phagocytic activity or respiratory burst of neutrophils (Jüttner et al., 2003) or even a decreased ROS production by HBOT (Kalns et al., 2002; Grimberg-Peters et al., 2016). The conflicting results could be explained by different methodologies: measurement of ROS (intracellular and/or extracellular), cell isolation and washing procedures, quantity of stimuli agents used (PMA, bacteria and zymosan etc.), the hyperbaric oxygen exposure time, fixation of cells, and time of examination post HBOT. Another explanation could be that the direct effect of HBOT on bacteria (see *Hyperbaric Oxygen, Pathogens and Antibiotics*) includes increased membrane instability and permeability (change in osmotic pressure) against the toxic reactive oxygen species (ROS) generated by both the neutrophils and HBOT.

Importantly, the viability and function of neutrophils are not adversely affected by intermittent exposure to elevated oxygen tensions. Neutrophils isolated from the peritoneal exudates of mice exposed intermittently to HBOT (100% O_2_, 2,5 ATA, 1.5 h twice daily for 8 days) showed the same ability to phagocytize and generate an effective respiratory burst (Gadd et al., 1990) after 16 sessions of HBOT compared to controls groups (non-HBOT, 10% O_2_ HBOT). In contrast to intermittent exposures, prolonged exposures, i.e. exceeding 24 hours, to normobaric hyperoxia seems to inhibit neutrophil function (Dunn and Smith, 1986). *In vitro* studies have also shown that phagocytosis was impaired by a 24 h exposure of HBOT (100% O_2_, 2.9 ATA (Weislow and Pakman, 1974).

### HBOT Reduce Neutrophil Adherence on the Endothelium

Important immune modulation during HBOT is the inhibition of neutrophil adhesion to vascular endothelium, which is why HBOT has been proposed as an adjunctive therapy in ischemia/reperfusion injury. This is of importance in IE, as neutrophils penetration in bacterial vegetations are limited and adherence to platelets trigger degranulation and neutrophil extracellular traps (NETs) contributing to additional platelets aggregation, vegetational growth and biofilm formation (Hsu et al., 2019). An experimental study by Zamboni et al. showed that following total ischemia of muscle flaps, the number of leukocytes adhering to vascular endothelium was significantly reduced by treatment with HBOT (Zamboni et al., 1993). The mechanisms responsible for reduced neutrophil sequestration have been reported in a series of elegant studies by Thom et al. (Thom, 1993; Chen et al., 1996). Focusing on the neutrophil modulation by HBOT, Thom et al. have shown that HBOT reduces neutrophil adhesion to a variety of substrates including fibrinogen. Using neutralizing antibodies, the effect has been shown to be specific to β2-integrin class of neutrophil adhesion molecules and is dose-dependent in the sense that exposure to pressures less than 2.4 ATA for 45 min has a negligible effect, whereas pressures of at least 2.8 ATA for 45 min completely inhibit adhesion, which is reversed by exposure to a stable cGMP analogue. The hypothesis that selective inactivation of β2-integrin function is sufficient to reduce ischemia/reperfusion (I/R) injury has been confirmed by other studies examining the efficacy of anti-Mac-1 monoclonal antibodies (Simpson et al., 1988; Zhang et al., 1995; Kalns et al., 2002). Overall, the intermittent exposure of HBOT used in clinically relevant regimes seems to have a positive effect on the neutrophil function.

However, other mechanisms may also play a part during HBOT. Buras et al. have shown that HBOT can reduce the expression of the neutrophil counter ligand ICAM-1 on vascular endothelium *in vitro* under conditions that mimic I/R (Buras and Reenstra, 2007). This implies that HBOT may target both the neutrophil and the vascular endothelium leading to a synergistic inhibitory effect, which might be of importance in IE by reducing the endothelial damage of the valves. Interestingly, a prospective study of 80 patients with necrotizing soft tissue infections (NSTI) receiving HBOT showed that HBOT might elevate soluble ICAM-1 (sICAM-1) and that the effect of HBOT was more pronounced in patients with NSTI and septic shock. Additionally, low baseline sICAM-1 was identified as an independent risk factor of 90-day mortality and baseline sICAM-1 was associated with severity of disease as indicated by significant correlations to simplified acute physiology score II. The authors speculate that HBOT might modulate endothelial shedding of ICAM-1 and reduce the inflammation on the endothelial line (Hedetoft et al., 2021).

### HBOT Reduce Neutrophil Inflammation

Reduction of endogenous damage-associated molecular patterns (DAMPs), the hosts “danger signal molecules” released upon infection and inflammation would be of great importance to dampen the exaggerated innate host response contributing to pathological cell damage seen in intravascular infections (**Figure 2**). An interesting study by Grimberg-Peters et al. revealed that HBOT could reduce the neutrophil extracellular traps (NETs) release from inflammatory neutrophils (Grimberg-Peters et al., 2016) and as mentioned earlier NETs are important in the progression of IE (Hsu et al., 2019). Various animal models indicate that neutrophil recruitment is significantly reduced by HBOT following I/R injury to the liver (Chen et al., 1998), intestine (Yamada et al., 1995; Tjärnström et al., 1999) and gracilis muscle (Zamboni et al., 1996). Other studies have shown that HBOT reduces the extent of tissue necrosis (Zamboni et al., 1989; Sterling et al., 1993; Liu et al., 2011) and lipid peroxidation (Thom, 1990) and maintains normal levels of tissue ATP (Haapaniemi et al., 1995). HBOT has also been shown to inhibit NF-κβ signaling pathways which are stimulated in various inflammatory and infectious diseases (Sakoda et al., 2004; Liu et al., 2018). Collectively, these studies indicate that HBOT ameliorate the collateral tissue damage and dampen proinflammatory responses.

Another interesting effect of HBOT is the mobilization of bone marrow derived stem/progenitor cells shown in humans and animals, primarily CD34^+^ cells (Thom et al., 2005; Milovanova et al., 2009). The mobilization of progenitor cells occurs *via* nitrite oxide-dependent mechanism. During infection, the turnover of host cells is high. Therefore, further recruitment of myeloid and endothelial progenitor cells might be very important in the healing of intravascular infections. Thus, higher levels of circulating endothelial progenitor cells (CD34^+^) could be beneficial to restore the lining of the valve endothelium and blood vessels (vasculogenesis) that are damaged during severe infections and IE.

## Effects of Hyperbaric Oxygen on Platelets, Endothelial Cells and Coagulation

### Direct Effects of HBOT on Platelet Aggregation and Adhesion

In the intact vasculature most platelets never undergo firm adhesion; however, upon vessel wall injury they rapidly adhere to the exposed extracellular matrix (ECM), become activated and form a platelet plug, thereby preventing blood loss and capillary leakage. In IE the same process can lead to excessive platelet aggregation on the valves contributing to expansion of vegetations (Lerche et al., 2019; Lerche et al., 2021).

There are sparse investigations of the direct effect of HBOT on platelets. In a study on isolated horse platelets Shaw et al. demonstrated that a single HBOT (100% oxygen, 2.2 ATA) had no detrimental effect on platelet biochemistry and did not cause overt oxidative stress (Shaw et al., 2005). In a subsequent study by Shaw et al. there were no significant difference in expression of surface markers (PECAM-1, CD62P or PAC-1) or nitrite oxide in HBO-treated compared to control-treated platelets. However, they noted an increased aggregation *in vitro* of platelet rich plasma stimulated with collagen, in the HBO-treated group compared to controls. However, the authors did not measure the two major receptors for platelets binding to collagen, the integrin alphaIIbetaI, Ig superfamily member and glycoprotein VI. Nor glycoprotein GPIb-V-IX was investigated, considered as an indirect collagen receptor acting *via* von Willebrand factor and essential for platelet/endothelial cell interactions with collagen at high shear rates (Clemetson and Clemetson, 2001; Pappelbaum et al., 2013). In addition, it was not investigated if HBOT modulates the surface adhesin of platelets. Translating *in vitro* platelet aggregation studies to *in vivo* conditions should be interpreted carefully due to easy mechanical activation of platelets *ex vivo*. More investigations are needed to clarify these aspects and the effect of HBOT of platelet-pathogen adherence in valve vegetations, which is of importance in IE (Kroh et al., 2009; Claes et al., 2017; Liesenborghs et al., 2019).

### Effects of HBOT on Platelet Chemotaxis

Platelets mediate leukocyte recruitment *via* two main mechanisms: (1) by serving as a docking site for immune cells along the endothelium surrounding the inflammatory focus and (2) through secretion of chemo-attractants. Studies have shown that platelets contain antimicrobial proteins in their granules (Yeaman et al., 1992), which for some infections can reduce the bacterial load (Sullam et al., 1993), while other pathogens are more resistant to these host antimicrobial peptides (Dhawan et al., 1997). Lerche et al. measured vascular endothelial growth factor (VEGF) in aortic valve vegetations and found it to be significantly decreased in HBOT-group compared with non-HBOT controls in an experimental *S. aureus* IE model (Lerche et al., 2017). VEGF is highly expressed in platelets (and activated endothelial cells), thus indicating that adjuvant HBOT reduced the platelets aggregation and endothelial inflammation on the valves (Lerche et al., 2017). This was also confirmed by the reduced weight and size of aortic vegetations in the HBOT group compared to control group. VEGF is tightly regulated by hypoxia-inducible factor (HIF)-1α, and HBOT might also modulate the expression of VEGF in platelets and endothelial cells, decreasing VEGF mRNA levels of these cells (Ferrara et al., 2003). Liu et al. have shown that pulmonary artery endothelial cells do not express VEGF under basal conditions; however, significant VEGF mRNA levels accumulate when these cells are exposed to hypoxia (Liu et al., 1995).

The oxygen sensitive modulation of HIF-1α expression and the downstream target genes (i.e. VEGF) is probably the main factor to regulate transcription of VEGF by host cells during HBOT. Timing of HBOT in hypoxic insult affects the transcription of HIF-1α and may lead to either an up-regulation or downregulation of HIF-1α (Amir and Shai, 2020). Another explanation for the reduced VEGF expression in infected valves (Lerche et al., 2017) could be the simple consequence of a more efficient treatment response, reducing the bacterial load and inflammation on the valves. The interplay between platelets and neutrophils are thoroughly reviewed elsewhere (Jenne and Kubes, 2015; Sørensen and Borregaard, 2016; Zucoloto and Jenne, 2019).

### Effects of HBOT on Endothelial Cells

It is well known that endothelial cells also perform hypoxia-induced exocytosis (Pinsky et al., 1996). Weibel–Palade bodies are secretory organelles in endothelial cells with high concentrations of von Willebrand factor (VWF), P-selectin, IL-8, IL-6, angiopoietin-2, and monocyte chemoattractant protein-1 alerting and recruiting platelets and neutrophils to the site of infection/inflammation. It is likely that HBOT could reduce this hypoxia-induced exocytosis of the endothelial cells, indirectly indicated by the decreased IL-8 and IL-6 observed in an experimental model of left-sided *S. aureus* IE (Lerche et al., 2017). Besides the virulence of pathogens, thrombin formation is also an important agonist to activate endothelial cells and platelets prompting the release of Weibel–Palade bodies content. In the experimental left-sided *S. aureus* IE model by Lerche et al. it has also been shown that by treating with adjunctive dabigatran and direct thrombin inhibitor the bacterial load and pro-inflammatory IL-8, IL-6, ICAM-1 and L-selectin of the valve vegetations were significantly reduced (Lerche et al., 2019), however VEGF levels were not affected. Combining different immunomodulatory targets together with antibiotic treatments would also be of great interest in future clinical studies of IE to personalize treatment regimens and improve clinical outcome.

## Hyperbaric Oxygen, Pathogens and Antibiotics

### Oxidative Stress on Pathogens

HBOT has a direct impact on pathogens, which is believed to be a result of formation of intracellular ROS as mentioned earlier. The ROS formation induced by the hyperoxia are toxic to the pathogen itself causing damage to DNA, proteins and lipids. Depending on the properties of the pathogen HBOT may have bactericidal (Walden and Hentges, 1975) or bacteriostatic effects (Korhonen, 2000). HBOT induces oxidative stress and eliminates microorganisms that lack antioxidant defense systems (McCord et al., 1971).

Oxidative stress has been suggested as a major factor in the development and progression of sepsis and septic shock (Motoyama et al., 2003; Ware et al., 2011). Hedetoft et al. measured oxidative stress markers by myeloperoxidase (MPO), two antioxidants superoxide dismutase (SOD) and heme oxygenase 1 (HO-1) and nitrite+nitrate in 80 patients with necrotizing soft tissue infections (NSTI) before and after HBOT. The study revealed an immediately increase in plasma MPO and SOD. In septic shock patient with NSTI, HO-1 was significantly increased after first HBOT. On the following day a significant reduction was observed for MPO and SOD and most pronounced for septic shock patients, highlighting the immediate immunomodulatory effect of HBOT. A high baseline SOD was associated with increased 90-day mortality (Hedetoft et al., 2021).

*S. aureus*, one of the dominating pathogens in IE, is well known for its adaptation to oxidative stress, where catalase activity in *S. aureus* is a key virulence factor. The involvement of oxidative stress in the susceptibility to antibiotics of *S. aureus* biofilm has recently been demonstrated by the protection against antibiotics provided by catalase (Haj et al., 2021). *S. aureus* has been intensively studied under HBOT. Bornside et al. showed that *S. aureus* growth could be retarded by 60% after 12 h of HBOT (Bornside, 1967). When cultures were transferred back to atmosphere condition exponential growth was restored. Importantly, they showed that the minimum inhibitory concentrations (MICs) for different antibiotics were reduced by HBOT, making the pathogen more susceptible to antibiotics (Bornside, 1967). In addition, Schwartz et al. showed that planktonic *S. aureus* exposed to 90 min of HBOT in a 4-h assay revealed a significantly lower colony forming units (CFU)/mL, compared to normobaric, normoxic growing *S. aureus* (Schwartz et al., 2021).

### Effects of HBOT on Bacterial Biofilms

HBOT is used as adjunctive treatment for patients with NSTI with virulent group A streptococcal infections (Siemens et al., 2016) also considered as an acute biofilm infection like IE. *In vitro* studies applying HBOT to bacterial biofilms have shown to augment the antimicrobial effect of ciprofloxacin (Kolpen et al., 2016; Kolpen et al., 2017). Furthermore, by knockout of the catalase gene in *Pseudomonas aeruginosa* (ΔkatA), the oxygen-dependent antibiotic killing is further enhanced (Kolpen et al., 2017). Another important aspect of HBOT, is that the oxygen penetration into the biofilm (*in vitro*) increases by four-fold, enhancing the metabolic activity and growth rate of the bacteria, thus, making them more susceptible to antibiotic treatment (Gade et al., 2018). Important studies by Durack et al. showed. that the *Streptococci*, at the peripheral border of vegetations were more metabolically active while those in the center were less active (dormant state), indicating oxygen tension gradients within the vegetations (Durack and Beeson, 1972a; Durack et al., 1973; Durack, 1975). This observation correlates well to the clinical situation and the biofilm architecture seen in valve vegetations, thus indicating that HBOT is an effective treatment option to reduce the bacterial load, size of valve vegetations and inflammation (Lerche et al., 2017).

### HBOT and Small-Colony Variants

The recalcitrant nature of IE can partially be explained by small-colony variants (SCVs) in the valve vegetations (Miller et al., 1978). SCVs of *S. aureus* are characterized by being non-hemolytic, having reduced coagulase activity and displaying non-pigmented colony morphologies 5–10 times smaller than the most common colony morphologies, due to auxotrophy (Proctor et al., 2006). These phenotypes are also characterized by increased antibiotics-tolerance [increased minimum inhibitory concentrations (MIC)] towards several antibiotic drugs (especially oxygen-dependent antibiotics) as well as increased catalase activity inducing tolerance to ROS. An important observation in experimental *S. aureus* IE is the formation of SCVs often seen in aminoglycoside treated animals (Lerche et al., 2015). This phenomenon is further induced by HBOT, as shown in an experimental *S. aureus* IE model of the cultured valve vegetations (Lerche et al., 2017). Sub analysis revealed that 54% in the HBOT group and 18% of control group had *S. aureus* SCVs in valve vegetations. The MICs of SCVs toward tobramycin and gentamicin were increased 50-fold compared to wild-type (0.125 μg/mL to 6 μg/mL). The increased oxidative stress on *S. aureus* both by HBOT and aminoglycoside influences *S. aureus* to change metabolism towards a more oxygen-independent metabolism. This fitness-cost of the pathogens, however, also impacts the virulence of the *S. aureus* SCVs, which are known for slow growth and reduced virulence compared to the wild-type (Proctor et al., 2014). This change in virulence was observed in the HBOT treated group, by significantly better clinical performance status compared to the control group (Lerche et al., 2017). This observation would also be relevant in a clinical protocol of HBOT in IE, highlighting the need to treat IE with a secondary antibiotic, which do not have an oxygen-dependent killing mechanism (i.e., rifampicin and linezolid), to prevent SCVs and with a good efficacy against biofilm (Lerche et al., 2021). This potential drawback of HBOT selecting these resistant bacterial phenotypes could, however, be limited by strategies by combining antibiotics with different modes of action. Several studies indeed suggest that combinations allow or an improved intracellular killing of SCVs, especially when they include rifampicin, linezolid or a highly bactericidal agent such as oritavancin (Baltch et al., 2008; Anh Nguyen et al., 2009; Lauridsen et al., 2012) If standard treatment length is followed IE the incidence of recurrent infection is very low. However, the strategy of combined antibiotic treatment (per oral, with different mode of actions) is already being used in standard treatment of stable IE patients with convincing results (Bundgaard et al., 2019; Iversen et al., 2019).

### Effects of HBOT on Pathogen Virulence

Studies have shown that HBOT also reduce α-toxin production in *Clostridium perfringens*. *Clostridium perfringens* growth is restricted at O_2_ tensions up to 70 mmHg, and α-toxin production is halted at tensions of 250 mmHg. The adaptation of *S. aureus* to SCVs is also accompanied by loss of the hemolytic toxin (α-, β-toxins) activity due to the oxidative stress induced by HBOT. These hemolysins are known to be of great importance in pathogenesis of IE and biofilm formation on the heart valves (Bhakdi et al., 1988; Chow et al., 1993; Bayer et al., 1997; Caiazza and O’Toole, 2003; Scherr et al., 2015). The impact of HBOT on other prevalent IE pathogens as hemolytic and non-hemolytic streptococci and enterococci remains to be investigated. Very rare cases of IE with anaerobe pathogens (*Cutibacterium agnes*, *Veillonella* and *Clostridium* spp.) are known to mainly affect prosthetic valves and a high portion of patients need cardiac surgery (Kestler et al., 2017), this group could also potential benefit of adjunctive HBOT regime to enhance the killing of the anaerobic pathogens and reduce the need for surgical intervention.

## Side Effects of Hyperbaric Oxygen Therapy

HBOT is generally accepted as a safe and non-invasive treatment option (Heyboer et al., 2017; Moon, 2019). Few absolute contra indications exist, among those with undrained pneumothorax. The most frequent and relatively benign side effects are middle ear barotrauma (in 2% of awake patients), which are reversible and can be prevented by autoinflation techniques or by paracentesis or inserting tympanostomy tubes. At high pressures and longer exposures oxygen has toxic effects with pulmonary and neurologic manifestations. However, pulmonary toxicity requires prolonged exposures to HBOT and is not a recurrent clinical problem (Hadanny et al., 2019). Oxygen seizures appear with an incidence of 0.01% depending on pressure and exposure time with no clinical evidence of long-term sequelae (Camporesi, 2014) and can be prevented by short air breathing periods during HBOT sessions (Hadanny et al., 2016a). Depending on the administration method applied (masks or hoods/monochamber), reversible myopia may appear (Hadanny et al., 2016b).

## Conclusion

Intermittent HBOT demonstrates multiple beneficial effects, dampening the detrimental host-pathogen interactions in IE. HBOT remains one of the most effective clinical means of oxygen delivery to deep vital tissue infections. There is a paucity of high-quality randomized controlled trials of HBOT, which makes it difficult to properly assess the clinical efficacy of HBOT. Hopefully, more well-designed trials are coming in the future. Patients with IE that would benefit from HBOT the most, would probably be the acutely ill with severe sepsis, large vegetations, dysregulated coagulation and thrombotic events, or until stabilization, or the patient groups which are not candidates for acute surgical intervention or removal of prosthetic valves and cardiac devices due to high risk of complications.

In conclusion, IE is a serious infectious disease and outcome data using present guidelines of antibiotic treatment of IE indicates the need for improved treatments strategies. HBOT is a promising candidate as an adjunctive treatment strategy due to the multifaceted effects and the pathophysiology of IE. HBOT as a potential treatment option in cases of IE is currently being studied in our center.

## Author Contributions

All authors listed have made a substantial, direct, and intellectual contribution to the work, and approved it for publication.

## Funding

CM was supported by the Novo Nordisk Foundation - “Borregaard Clinical Scientist Grant” (Grant No. NNF17OC0025074). OH was supported by the projects of PERMIT and PERAID (grant number 8113-00009B and 8114-00005B) funded by Innovation Fund Denmark, EU Horizon 2020 (ERA Permed project 2018-151) and Nordforsk (project no. 90456).

## Conflict of Interest

The authors declare that the research was conducted in the absence of any commercial or financial relationships that could be construed as a potential conflict of interest.

## Publisher’s Note

All claims expressed in this article are solely those of the authors and do not necessarily represent those of their affiliated organizations, or those of the publisher, the editors and the reviewers. Any product that may be evaluated in this article, or claim that may be made by its manufacturer, is not guaranteed or endorsed by the publisher.
